# Deep-Ultraviolet AlN Metalens with Imaging and Ultrafast Laser Microfabrication Applications

**DOI:** 10.1021/acs.nanolett.4c05552

**Published:** 2025-01-29

**Authors:** Yu Chieh Peng, Yu Jie Wang, Kuan-Heng Chen, Yu Hung Lin, Haruyuki Sakurai, Hsueh-Chih Chang, Cheng-Ching Chiang, Ruei-Tzu Duh, Bo-Ray Lee, Chia-Yen Huang, Min-Hsiung Shih, Ray-Hua Horng, Kuniaki Konishi, Ming Lun Tseng

**Affiliations:** † Institute of Electronics, National Yang Ming Chiao Tung University, Hsinchu, 30010, Taiwan; # Institute for Photon Science and Technology, The University of Tokyo, Tokyo 113-0033, Japan; § Electronic and Optoelectronic System Research Laboratories, Industrial Technology Research Institute, Hsinchu, 30010, Taiwan; ∥ Department of Photonics, National Yang Ming Chiao Tung University, Hsinchu 30010, Taiwan; ⊥ Research Center for Applied Sciences, Academia Sinica, Taipei 11529, Taiwan

**Keywords:** metasurface, metalens, deep ultraviolet light, laser direct writing, DUV imaging

## Abstract

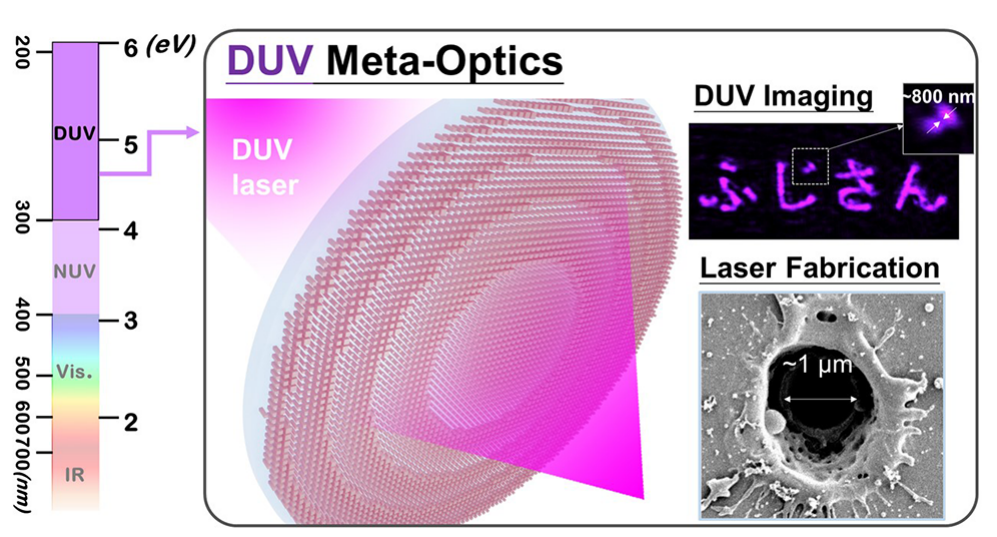

Deep-ultraviolet (DUV) light is essential for applications including fabrication, molecular research, and biomedical imaging. Compact metalenses have the potential to drive further innovation in these fields, provided they utilize a material platform that is cost-effective, durable, and scalable. In this work, we present aluminum nitride (AlN) metalenses as an efficient solution for DUV applications. These metalenses, with a thickness of only 380 nm, deliver DUV focusing and imaging capabilities close to the theoretical diffraction limit. Leveraging their robustness to intense ultrafast laser irradiation, we demonstrate successful DUV ultrafast laser direct writing of microstructures on a polymer film and silicon substrate. These results underscore the significant promise of advancing photonic technologies in this critical wavelength regime.

Metasurfaces,^1−3^ two-dimensional artificial nanostructures, have revolutionized numerous photonic applications. By carefully engineering resonant unit cells^4,5^ and selecting appropriate constituent materials, metasurfaces overcome the limitations of conventional photonic devices, driving innovation for applications including light manipulation^6−12^ and generation,^13−16^ imaging,^17−20^ and biosensing.^21−27^ A key area where metasurfaces can offer significant innovation is the deep ultraviolet (DUV) regime, with wavelengths from 200 to 300 nm (from 6.2 to 4.1 eV). DUV light plays a crucial role in advanced fabrication processes for photonic and electronic devices,^28,29^ as well as biomedical spectroscopy,^30−200^ label-free molecular mapping,^34,35^ and clinical diagnosis.^35^ Recent works have shown the promising potential of metasurfaces in DUV nanophotonics. Functional metasurfaces that enhance DUV light harvesting^36^ and generation^37^ have been demonstrated. Leveraging their high design flexibility, metasurfaces can enable further the realization of novel miniaturized DUV elements. Traditional optical devices rely on gradual light modulation as light propagates through the material, but they face limitations when materials with the necessary optical transparency or mechanical strength for reliable operation and fabrication are unavailable. Common optical materials, such as general glasses,^38^ exhibit significant absorption below 300 nm. Conventional DUV components rely on specialized materials (e.g., UV-grade fused silica, MgF_2_) or sophisticated reflection-based designs, leading to higher production costs and bulkier equipment. Although high-performance DUV components like objective lenses are available, they remain expensive and may not be suitable for some applications requiring compact, scalable, or integrated solutions. Miniaturized DUV devices offer a promising solution to these challenges and are important for advancing next-generation DUV photonics.

Introducing metasurface optics (meta-optics) to the DUV regime can effectively address several challenges. Metasurfaces achieve precise light control through engineered resonances in their unit cells, allowing for ultrathin designs and enabling fabrication by nanopatterning low-loss thin films. To realize efficient DUV dielectric metasurfaces, a critical requirement is finding commonly available dielectric materials with suitable properties. For effective light modulation, the constituent material of the metasurfaces should have a high refractive index (*n* > 2) and a low extinction coefficient (*k* ∼ 0). Previous works use diamond^39^ or compound dielectrics such as hafnium dioxide (HfO_2_),^40^ and zirconium dioxide (ZrO_2_)/polymer mixture^41^ to fabricate DUV metalenses and meta-holograms. However, preparing high-quality HfO_2_ and diamond films and nanostructures typically requires sophisticated film deposition techniques. Polymer-based metasurfaces, while offering some advantages, may face stability challenges under intense laser power or significant temperature fluctuations. Additionally, the potential of DUV metasurfaces in critical applications such as imaging and laser fabrication remains largely unexplored.

Among potential materials, AlN stands out as a unique solution for DUV meta-optics due to its distinct advantages. It is cost-effective due to the abundance of its earth-abundant constituent elements, and offers excellent thermal conductivity (κ = 285 W/(m·K)), a low expansion coefficient (∼4.5 × 10^–6^/°C), and a suitable thermo-optic coefficient^42^ ( at 266 nm). These properties make AlN ideal for nanophotonic applications requiring high power, such as nonlinear optics and laser fabrication in the DUV regime. Furthermore, as AlN has been widely used in integrated circuits and memory devices for years, the film deposition techniques and high-throughput fabrication processes have been well-established. Despite this, experimental demonstrations of AlN metasurfaces in the DUV range have been limited.

Here, we present the experimental realization of AlN metalenses and their applications in the DUV regime, achieving both imaging and laser direct writing. These metalenses deliver precise light focusing near the theoretical diffraction limit and enable DUV imaging with submicron resolution. Furthermore, the robustness of the AlN metalenses allows for effective integration into DUV ultrafast laser direct-write systems, facilitating the fabrication of microstructures on polymer films and silicon substrates under intense laser power. This combination of ultracompact device size, precise DUV focusing, and compatibility with standard semiconductor manufacturing processes highlights the potential of AlN for advancing miniaturized DUV photonic systems.

## Design and Fabrication

**Figure 1:**
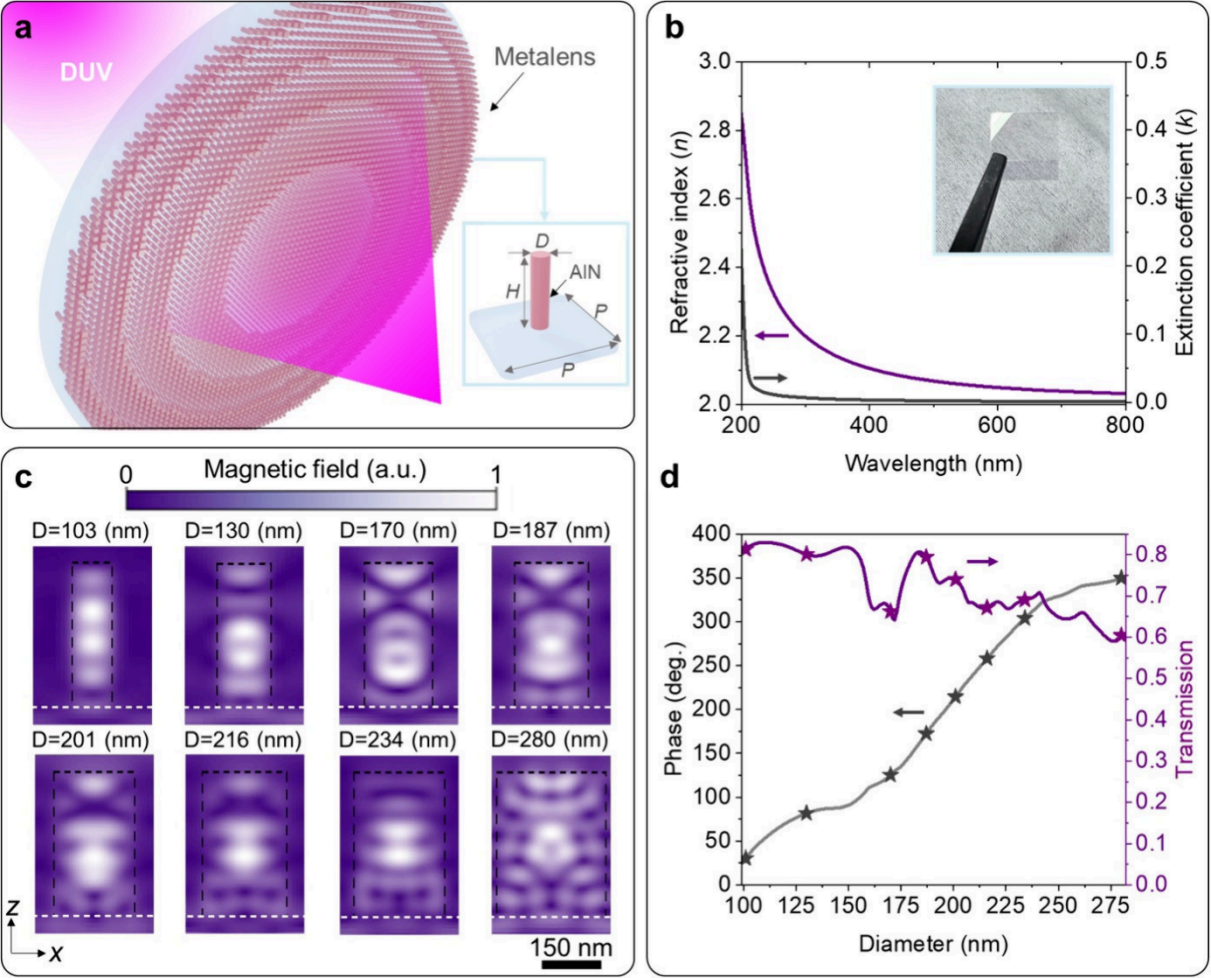
DUV AlN metalens. (a) Schematic of the DUV metalens. Inset: The unit cell of the metalens. *P*: 300 nm. *H*: 380 nm. *D*: Diameter, ranging from 100 to 280 nm. (b) Optical constants of the AlN film used in this paper. Purple line: refractive index. Black line: extinction coefficient. Inset: Optical image of the AlN film. (c) Simulated magnetic field distribution of the nanopillars used in the metalens designs. In each panel, the black dashed line indicates the boundary of the AlN nanopillar, while the white dashed line indicates the surface of the substrate. (d) Transmittance and phase of the nanopillars of different diameters (labeled as *D* in (a)).

Figure 1a shows a schematic of the reported metalenses, which consist of arrays of circular AlN nanopillars (parameters shown in the inset of Figure 1a) on sapphire substrates. The symmetric geometry of the unit cells ensures the versatility of different polarized light sources. To design the geometric parameters of the nanopillars for the DUV metalenses, we used commercial FDTD-based software Lumerical to simulate and analyze their properties. In the simulation, we imported the optical constants of the AlN film deposited on a sapphire substrate into the software. The optical constants were experimentally measured from the AlN film (the inset in Figure 1b) by using an ellipsometer, as plotted in Figure 1b. The experimental refractive index and extinction coefficient of the AlN film used in this work are ∼2.2 and ∼0 beyond 230 nm, respectively. The proper refractive index value and low optical loss confirm AlN’s suitability as the constitution material of metasurfaces in the DUV range. The working mechanism of the reported metalens relies on local phase modulation induced by low-quality-factor resonances excited in the individual AlN nanopillars.^1^Figure 1c shows the simulated magnetic field distribution for nanopillars of varying diameters. As seen in the panels of Figure 1c, the magnetic field is majorly localized within the AlN nanopillars. Additionally, different distributions of the field antinodes and nodes in the nanopillars are observed, confirming that the nanopillars exhibit distinct resonance modes at 266 nm. Figure 1d shows the simulated transmittance and phase modulation of nanopillars with different diameters at 266 nm. The nanopillars are arranged in a square lattice. Linearly polarized light incident from the substrate side is used in the simulation. As shown in Figure 1d, a full 2π phase coverage can be achieved by using the nanopillars. We carefully selected eight nanopillars for the metalens designs to ensure proper device transmittance. The selected nanopillars show overall transmittance larger than 70%. In the metalens designs, we used the hyperbolic phase profile *ϕ(*x, y) for planar lenses to calculate the phase distribution:^43,44^
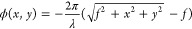
1where *f* is the focal length, and λ is the incident wavelength (266 nm). By changing the nanopillars’ diameter along the surface as the function of their position (*x*, *y*), the required phase distribution can be subsequently encoded.

## Nanofabrication and Characterization

**Figure 2:**
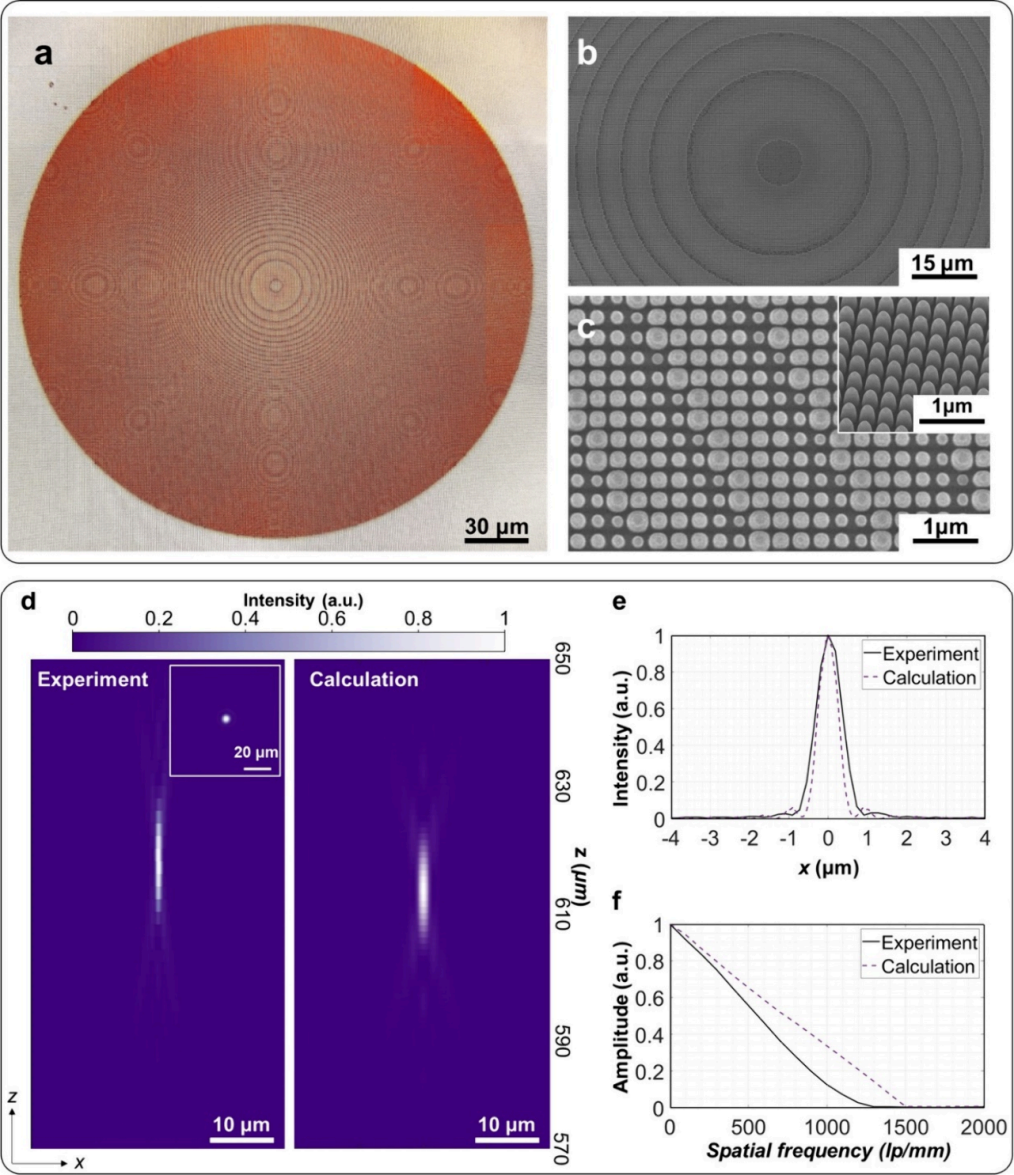
Fabrication and characterization of the DUV metalens. (a) Optical image of the metalens. (b–c) SEM images of the metalens. Inset of (c): The titled SEM image. (d) Left panel: experimental focusing profile. Inset: Image of the focal spot. Right panel: Calculated focusing profile. (e) The line spread function for the focal spot of the metalens. Solid line: experimental data. Dashed line: calculated data. (f) MTF of the metalens. Solid line: experimental data. Dashed line: calculated data. lp/mm: line pairs per millimeter.

For the experimental implementation of the metalenses, we performed the fabrication process consisting of electron beam lithography, hard mask deposition, and dry etching on an AlN film (see Methods section and Supporting Information). Figure 2a shows an optical microscopic image of the fabricated metalens with a numerical aperture (NA) of 0.2, while the corresponding scanning electron microscopic (SEM) images are presented in Figures 2b and 2c. The results confirm the successful fabrication of the AlN metalens. To characterize its DUV focusing capability, we built an imaging system following approaches commonly used in previous studies.^18,45^ A schematic and detailed description of the setup are provided in Figure S1 of the Supporting Information. The measurement result of the metalens’ DUV three-dimensional focusing profile is presented in the left panel of Figure 2d. A focal spot of the 266 nm laser is clearly observed at *z* = 618 μm. The image of the focal spot (inset of Figure 2d.) confirms the highly symmetric shape of the focal spot produced by the metalens. The metalens generates a focal spot with a full with half-maximum (FWHM) of 0.75 μm (Figure 2e,) with a Strehl ratio (SR) of 0.75. The result of the focal spot analysis is very close to the theoretical value of the diffraction limit (0.66 μm). Additionally, we simulated the focusing profile using calculations based on the beam propagation method (detailed in Supporting Information) and presented the results in the right panel of Figure 2d. The metalens shows a focal length slightly longer than the theoretical value. To assess the optical quality of the reported AlN metalenses, we further estimated the modulation transfer function (MTF)^46,47^ of the reported metalens and compared the experimental and calculated results. The MTF quantifies the system’s contrast reproduction at different spatial frequencies and can be obtained by applying a Fourier transform to the line spread function (Figure 2e) of the focusing profile. As shown in Figure 2f, the experimental MTF retrieved from the line spread function of the focal spot exhibits a cutoff spatial frequency that is close to, but slightly smaller than, the cutoff spatial frequency of the theoretical result. Overall, the fabricated metalens shows light focusing properties close to the theoretical prediction. The discrepancy between the theoretical and experimental results may be attributed to imperfections in the sample introduced during fabrication. Additionally, high-NA DUV metalenses can be achieved. As demonstrated in Figure S2 in Supporting Information, by modifying the nanopillars’ parameters, a DUV metalens with an NA of 0.8, which is considered challenging to realize with conventional optics, can be achieved. These results confirm the potential of the proposed design approach for developing advanced DUV components. We also verified that the metalens shows a proper tolerance to the sample imperfection, see the discussion in Supporting Information.

## DUV Imaging

**Figure 3:**
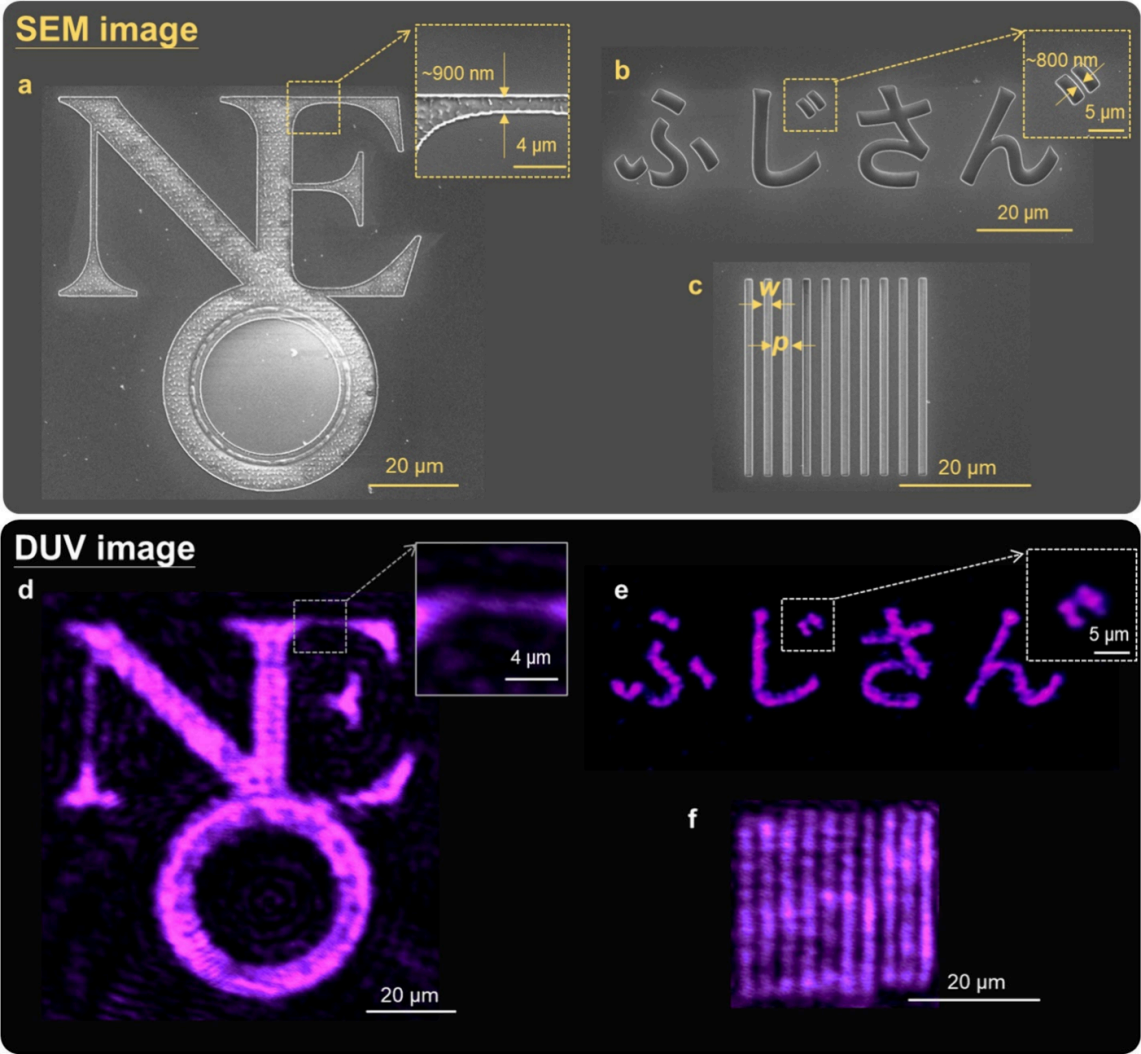
DUV Imaging of patterns with sub-μm features. (a–c) The SEM images of the patterns of (a) overlapping “NEO”, (b) Japanese letters (Fuju mountain), and (c) Al slits. *p*: 3 μm, *w*: 1 μm. Insets in (a) and (b) are the magnified SEM images of the highlighted regions. (d–f) Corresponding transmissive DUV images of the patterns produced by the DUV metalens. Insets in (d) and (e) are the magnified SEM images of the highlighted regions.

We further validate the imaging capability of the proposed metalens. DUV imaging is critical for applications in biomedical imaging,^48^ clinical diagnostics,^48,49^ and environmental monitoring.^50^ For imaging, we fabricated a metalens with a 1 mm diameter and an NA of 0.2. To assess imaging performance, we used focused ion beam milling to create patterns in a 50 nm-thick aluminum (Al) film deposited on a UV-grade silica substrate. The patterns included overlapping letters “NEO” (Figure 3a), Japanese characters (representing Mount Fuji, Figure 3b), and a slit array (1 μm in width, 3 μm in period, Figure 3c). The observation was performed in transmission mode. During imaging, the patterned Al film was illuminated with the 266 nm laser from the substrate side. The sample was positioned at one to two times the focal length of the metalens, producing a magnified image. The distance between the metalens and the combination of the DUV objective silica lens after the metalens were carefully adjusted to expand and project the image to the UV camera. The schematic of the setup can be found in Supporting Information. Figures 3d–f display the captured DUV images, clearly showing the metalens’ successful imaging of the patterns. Minor distortions are observed, likely due to laser diffraction from the slits and aberrations in the optical setup between the metalens and the camera. However, the metalens effectively resolves fine features, including the details of the letter “E” (insets of Figure 3a and 3d), the Japanese characters (insets of Figure 3b and 3e), and the slits, with a resolution of approximately 800 nm, very close to the theoretical diffraction limit associated with the NA of the metalens (∼665 nm) used in the measurement. Some areas of Al within the patterns were not completely removed by the ion beam, leaving residual Al nanostructures in the slits. These remnants may partially block incident DUV light, causing parts of the patterns to appear unclear in the transmission image (*e.g*., the middle section of the letter “E”). Overall, these results confirm the imaging capability of the reported DUV metalens.

## High-Temperature Tolerance

**Figure 4:**
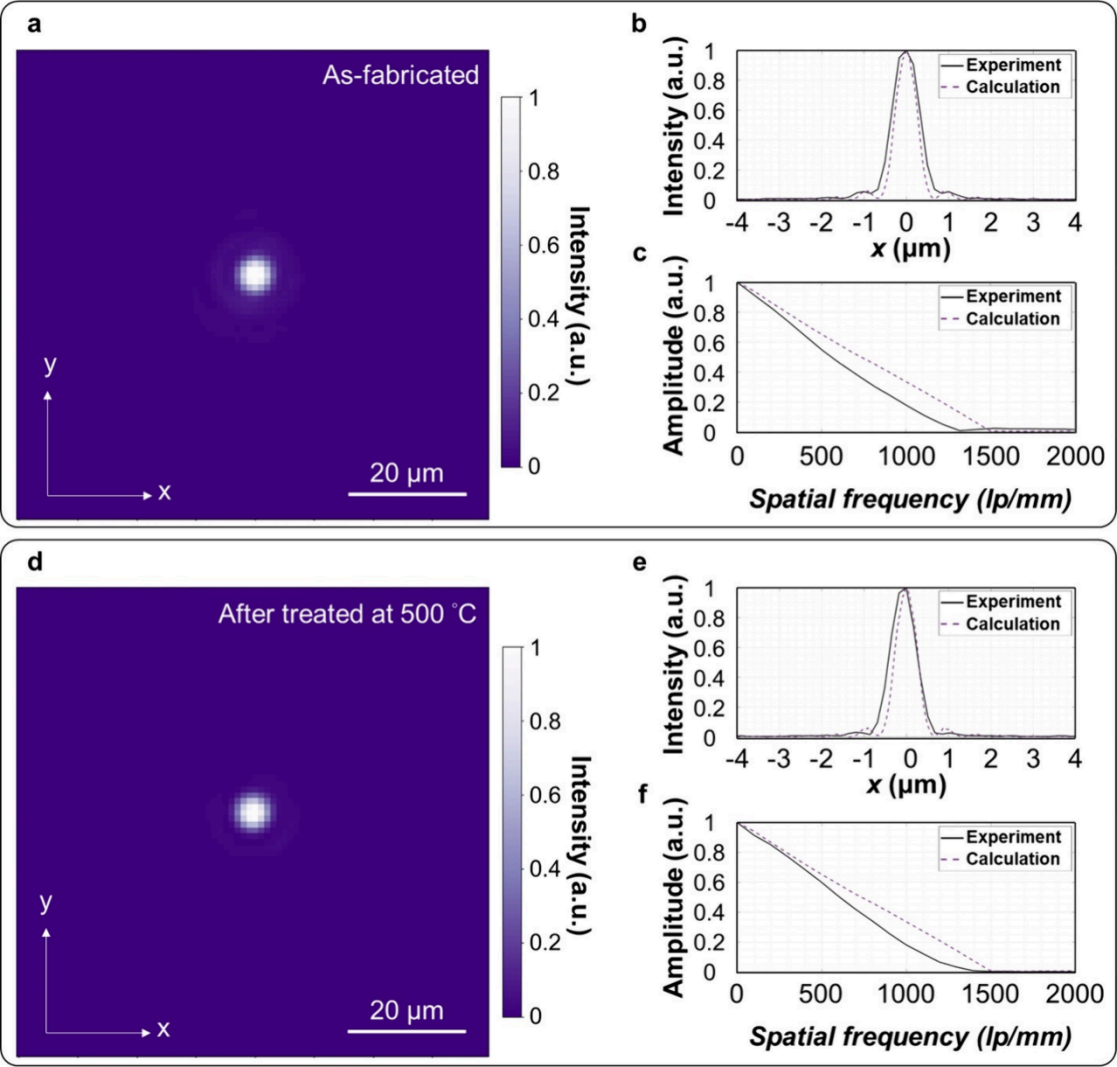
Tolerance to high temperature. (a) The focal spot of the as-fabricated metalens (NA: 0.2, Diameter: 250 μm) and the corresponding (b) line spread function and (c) MTF analysis. (d) focal spot of the metalens after the thermal treatment at 500 °C and the corresponding (e) line spread function and (f) MTF analysis. Scale bar in (a) and (d): 20 μm. lp/mm: line pairs per millimeter.

Understanding the tolerance of the reported AlN metalenses under harsh operating conditions will be helpful for evaluating their practicality to applications that would meet high temperature changes. Due to AlN’s high melting point and thermal stability,^51^ the reported metalenses are expected to withstand elevated temperatures. To test this, we fabricated a metalens (NA: 0.2, diameter: 250 μm) and heated it at 500 °C in an argon-filled furnace for 1 h. Its focusing performance was evaluated before and after heating. Figures 4a-c show the metalens’ focal spot and the associated analysis of the line spread function and MTF, while Figures 4d-f show the corresponding results for the metalens after the treatment. The FWHM (∼0.75 μm) and the MTF curve of the metalens before and after the treatment remain almost identical, indicating no apparent performance degradation after the high-temperature treatment. We further heated the metalens at 1000 °C, performed the measurement, and confirmed the metalens still provides proper DUV light focusing after such a temperature treatment, see Figure S3 and the discussion in Supporting Information. The results demonstrate the great thermal stability of the AlN metalens.

## Ultrafast Laser Direct Writing

**Figure 5:**
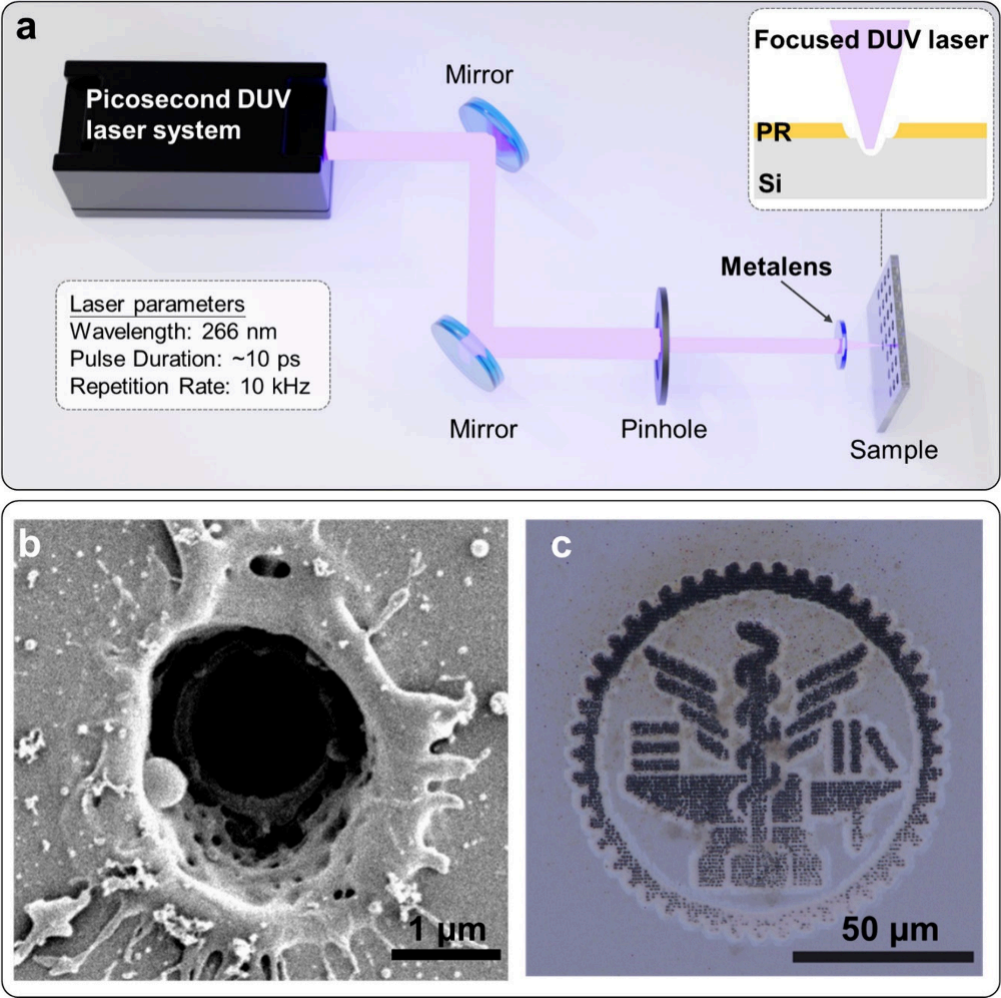
Laser direct writing with the DUV AlN metalens. (a) Schematic of the experimental setup for laser direct writing. Inset: The side view of the fabricated structure made by the DUV laser. PR: photoresist; Si: silicon. (b) SEM image of a laser-produced crater on a photoresist-coated silicon sample by 10-pulse DUV picosecond pulse irradiation using the AlN metalens. (c) Optical microscopic image of the National Yang Ming Chiao Tung University logo fabricated on the photoresist-coated silicon sample. The logo was used with the permission from National Yang Ming Chiao Tung University.

To further showcase the application potential of the reported AlN metalenses, we demonstrate their feasibility for ultrafast laser direct writing applications. The experimental setup is shown in Figure 5a. The light source was a picosecond DUV laser system (LDH-X0810, Spectronix). The central wavelength was 266 nm, and the pulse duration was approximately 10 ps. The laser was operated at a 10 kHz repetition rate at approximately 180 mW average power. This light was cut to a diameter of 800 μm by a pinhole. The average power after the pinhole was 6.3 mW, corresponding to a pulse energy of 630 nJ. The laser was subsequently sent to a metalens with a diameter of 750 μm and focal distance of 2 mm (NA 0.184). The focused light was irradiated onto a sample consisting of a 1.3-μm-thick layer of photoresist (JSR 7790G) coated on a silicon substrate. This sample was mounted onto an automated three-dimensional stage (OSMS20–35(XYZ); OptoSigma) for micropositioning.

A SEM image of the result of irradiating ten DUV pulses onto the sample is shown in Figure 5b. It can be seen that the pulsed-DUV laser not only ablates the photoresist (large outer circle surrounded by melt), but also creates a hole in the silicon substrate below, which is visible as the black central hole in the crater bottom (a schematic is plotted in the set of Figure 5a). The diameter of the ablated resist is about 2 μm and the diameter of the hole in the silicon is 1.5 μm. It is clearly shown that with our DUV metalens, near-perfectly circular micrometer-scale holes can be produced in the silicon substrate. We note that producing such Si microholes by DUV light often requires objective microscope lenses, which, while available, are less common in the DUV region compared to the visible range. Repeated experiments over a couple of hours showed no discernible degradation of fabricated features, suggesting reasonable durability of the fabricated metalens to continued DUV irradiation, even at ablative intensities. By precisely moving the three-dimensional stage, patterns could also be fabricated with our simple setup. Figure 5c shows the fabrication result of the National Yang Ming Chiao Tung University logo^52^ on the same photoresist-coated silicon substrate. The logo was rasterized into a 100 × 100-pixel image from which the patterning coordinates were extracted. These coordinates were processed onto the sample at a scaling factor of 1 μm per pixel. Here, a total of 3357 points were processed, with 100 pulses irradiated at each processing point. Due to some sample tilt and the resultant focal point drift, ablation was stronger along the top side of the sample than on the bottom, but otherwise, the logo can be seen to be nicely fabricated. The results suggest that our metalens is suitable for performing laser direct writing on polymer films and semiconductor materials, pointing out the metalens’ great potential for efficient fabrication of novel photonic and electronic devices.^28,53^ In addition, the intensities used in this experiment were sufficient to ablate the material, representing the upper power limit typically required for various nondestructive applications, such as sensing, imaging, and photolithography. Therefore, our metalens technology is practical for a wide range of DUV applications.

In conclusion, we have experimentally demonstrated that AlN metalenses offer a feasible platform for DUV nanophotonics, combining precise light focusing and near-diffraction-limited imaging with exceptional tolerance for high temperature. The robustness of AlN metalenses further enables their application in ultrafast laser direct writing for micropattern fabrication. Our findings highlight the strong potential of AlN metalenses and metasurfaces for a range of innovative applications. For instance, since most biomolecules exhibit strong absorption^30^ in the DUV spectrum, integrating AlN metasurfaces with functionalities such as imaging, focusing, and polarization control into a compact system will allow for label-free molecular mapping^34^ of biological tissues and cells. Furthermore, the design flexibility of metalenses offers exciting opportunities to enhance the functionality of the reported metalenses. Strategies such as superimposing multiple phase profiles^54^ or stacking metalenses^55^ could be explored to address off-axis aberrations. Tailoring the unit cell designs could enable functionalities like chromatic aberration correction^56^ or higher numerical apertures.^57^ Additionally, emerging approaches such as artificial-intelligence-driven inverse designs^58,59^ hold significant promise for further optimizing the performance of DUV metalenses. Due to the compact size and integration flexibility, the reported metalenses hold potential for incorporation into laser scanning imaging systems^57^ for diverse DUV imaging applications in the future. Incorporating the ultracompact AlN metalenses into optoelectronic devices, such as DUV micro-light-emitting diodes (DUV micro-LEDs)^60,61^ can further bring innovations for the functionality and performance. While structured light holds great promise for advanced DUV laser nanofabrication, its implementation remains challenging due to the scarcity of suitable components. By leveraging the knowledge of recent demonstrations,^8,62^ novel DUV beam profiles can be realized to address this challenge and enable new systems. Supported by recent advancements in CMOS-compatible, large-scale metasurface fabrication,^63−65^ this work establishes a promising pathway toward high-throughput production of DUV metasurfaces, paving the way for further advancements in DUV photonic technologies.

## Methods

### Unit Cell Design

We employed Lumerical, an FDTD-based simulation software, to design and analyze the metasurfaces presented in this study. Periodic boundary conditions were applied along the *x*- and *y*-axes (defined in Figure 1c), while perfectly matched layer (PML) boundary conditions were set in the *z*-direction. The incident light was incident from the substrate side.

### Sample Fabrication

A schematic of the fabrication process flow is presented in Figure S4 in Supporting Information. The 380-nm-thick AlN film was deposited on a *c*-plane sapphire substrate via plasma-enhanced chemical vapor deposition (PECVD). We chose sapphire as the substrate material because it has high transparency to DUV light and exhibits proper lattice matching with AlN, which is key for the film deposition process. The thin film shows a surface roughness of ∼0.6 nm in the atomic force microscopy measurement (Figure S5 in Supporting Information)). A 200-nm-thick silica layer, serving as the etching mask, was deposited on the AlN surface. A 90-nm-thick layer of poly(methyl methacrylate) (PMMA 950 A2, from MicroChem) electron-beam resist was spin-coated on the sample, followed by coating a water-soluble conductive polymer ESPACER layer (Resonac) for surface charging dissipation. Electron beam exposure was conducted using a Raith Voyager e-beam writer at 50 kV and 100 pA. After exposure, the development process was carried out in a MIBK/IPA solvent for 75 s. A 30-nm-thick Cr layer was then evaporated onto the sample, and lift-off in acetone prepared the metal hard mask for subsequent dry etching. The pattern was transferred to the AlN layer using inductively coupled plasma reactive ion etching (ICP-RIE). First, a gas mixture of SF_6_, CH_2_F_2_, He, and N_2_ etched the SiO_2_ layer, followed by BCl_3_ and Cl_2_ to etch the AlN film. Finally, the remaining silica atop the metalenses was removed with 1% HF solution. For the fabrication of the micropatterns used to verify the imaging capability of the DUV metalens, a 50 nm-thick Al film was coated on a cleaned silica substrate by using an electron beam evaporator. A focus ion beam system (Helios 660 NanoLab, FEI Company) was used to make the patterns on the thin film. The beam current of the gallium ion beam was set to 1 nA with an acceleration voltage of 30 kV.

### Film Preparation for Laser Direct Writing

The photoresist (JSR 7790G, from JSR Corp.) was spin-coated onto a cleaned silicon substrate at 3000 rpm for 30 s and baked at 110 °C for 90 s. The thickness of the photoresist layer on the silicon was 1.3 μm.

## Supporting Information Available

Measurement setup; simulating DUV focusing of the AlN metalens; DUV focusing simulation of a high-NA AlN metalens; SEM image of a laser-produced crater on a photoresist-coated silicon; comparison between the DUV objective and metalens; tolerance of the AlN metalenses for the fabrication imperfections; DUV focusing of the AlN metalens after thermal treatment; fabrication process flow; AFM image of the AlN film (PDF)
Optical constants of AlN (XLSX)
