# Supplementary material for: Deep-Ultraviolet AlN Metalens with Imaging and Ultrafast Laser Microfabrication Applications
Source: Nano Lett. 2025 Jan 29;25(8):3141–9. doi: 10.1021/acs.nanolett.4c05552 (PMC11869270; doi:10.1021/acs.nanolett.4c05552)
Supplement: Supplementary file 1 — nl4c05552_si_001.pdf [file nl4c05552_si_001.pdf]

Supporting information for "Deep-Ultraviolet AlN Metalens with Imaging and Ultrafast Laser Microfabrication Applications"

Yu Chieh Peng[1‡], Yu Jie Wang[1‡], Kuan-Heng Chen[1‡], Yu Hung Lin[1], Haruyuki Sakurai[2], Hsueh-Chih Chang[1,3], Cheng-Ching Chiang[1], Ruei-Tzu Duh[1], Bo-Ray Lee[1], Chia-Yen Huang[4], Min-Hsiung Shih[5], Ray-Hua Horng[1], Kuniaki Konishi[2], and Ming Lun Tseng[1*]*

[1] The Institute of Electronics, National Yang Ming Chiao Tung University, Hsinchu, 30010, Taiwan

[2] Institute for Photon Science and Technology, The University of Tokyo, Tokyo 113-0033, Japan

[3] Electronic and Optoelectronic System Research Laboratories, Industrial Technology Research Institute, Hsinchu, 30010, Taiwan

[4] Department of Photonics, National Yang Ming Chiao Tung University, Hsinchu 30010, Taiwan

[5] Research Center for Applied Sciences, Academia Sinica, Taipei 11529, Taiwan

**Corresponding authors' email: kkonishi@ipst.s.u-tokyo.ac.jp (K.K.); mltseng@nycu.edu.tw (M.L.T.)*

List of Supporting Information

I. Measurement setups
II. Simulating DUV focusing of the AlN metalens
III. DUV high-NA AlN metalens: simulation demonstration
IV. DUV focusing of the AlN metalens after thermal treatment
V. SEM image of a laser-produced crater on a photoresist-coated silicon
VI. Comparison between the DUV objective and metalens
VII. Tolerance of the AlN metalenses for the fabrication imperfections
VIII. Fabrication process flow
IX. AFM image of the AlN film

I. Measurement setup

As shown in **Figure S1a**, a 266-nm continuous wave (cw) semiconductor laser (CryLaS, FQCW266-50) is used as the light source. A spatial filter consisting of a pair of fused silica lenses (UV-Grade Fused Silica Lens, Thorlabs) and a pinhole (diameter: 200 μm) was used to purify the laser profile. A tunable neutral-density filter (NDM2, Thorlabs) was used to control the incident laser power. We used a pinhole to control the laser beam size. The sample was mounted on a three-dimensional linear stage (Newport). For recording the imaging, a 4-f system (consisting of a UV objective (Mitutoyo M Plan UV 80X, NA:0.55) lens and a fused silica tube lens (Thorlabs, LA4148, NA: 0.25) and a UV camera (iDule, ID8MUVS-CL) were mounted on a motorized linear stage that can move along the z-axis (OSMS20-35(x)). A filter was placed in front of the UV camera to filter out the possible photoluminescence generated from the optical components under DUV illumination. In the measurement, in the beginning, the focal plane of the imaging system was placed on the metalens's surface. The focal plane was gradually moved away from the metalens sample. Meanwhile, the UV camera recorded the DUV profile at the different z coordinates. The three-dimensional focusing profile was then reconstructed by analyzing the serial images. The setup allows for the efficient characterization of the three-dimensional focusing profile of the metalens, as is standard in the literature. In addition, using a confocal microscopy setup to characterize the metalens' focusing capability is another possible approach[1].

A schematic of the setup used for the imaging is presented in **Figure S1b**. The configuration of setup is similar to the one used in other previous works[2, 3]. Due to the small size of the metalens, the produced image could be too small for a clear visualization by using the DUV camera. In the measurement, the 80× objective was replaced with a 20× objective (Mitutoyo, NA: 0.36). The image produced by the metalens was collected and expanded by the 20× objective and projected to the camera by the silica lens. We note that for an imaging system consisting of multiple lenses, the resolution is majorly determined by the lens with the smallest NA value as it determines the system's cutoff spatial frequency. For our experiment, the NA of the metalens is smaller than that of the UV objective (NA:0.36) behind it.

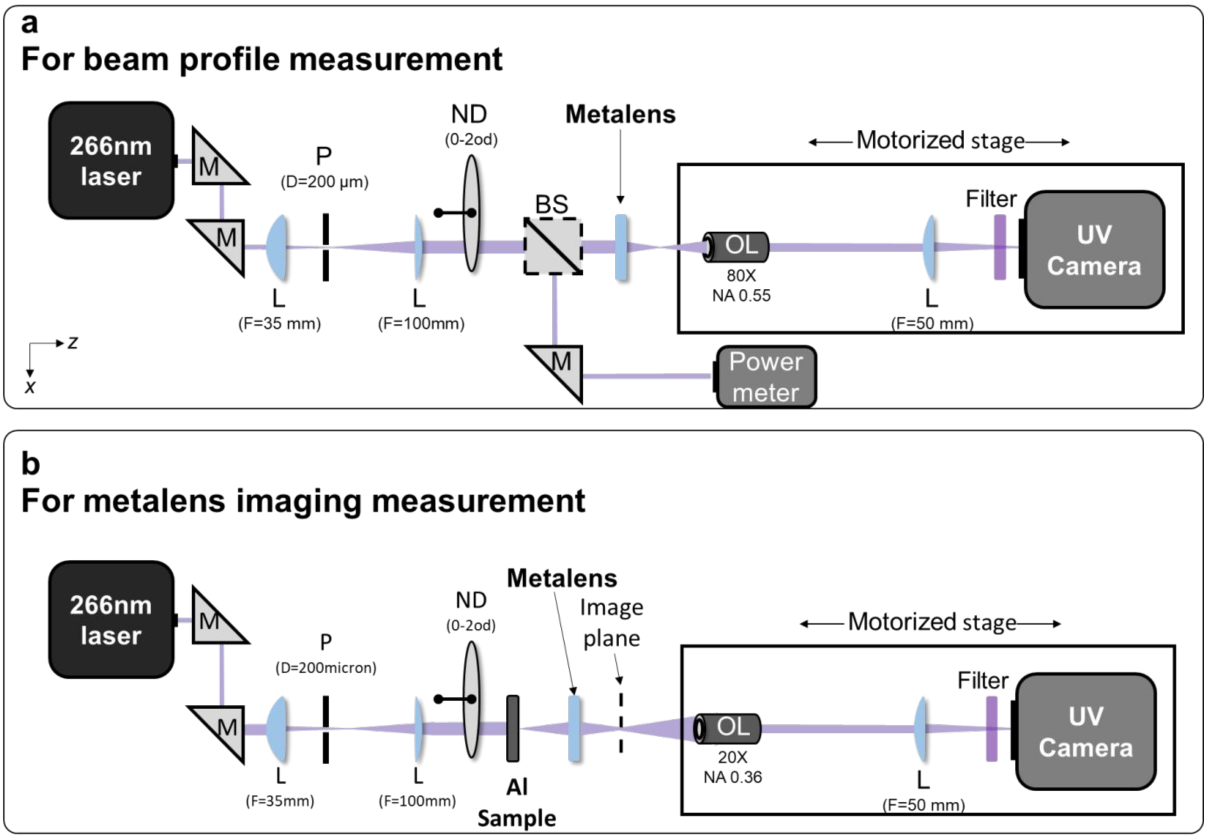

Figure S1. Characterization setups. (a) Setup for measuring the focusing profile. (b) Setup for measuring imaging produced by the DUV metalens. The light path behind the sample represents a light path from the point source on the sample. M: aluminum mirror; L: Fused silica lens; P: Pinhole; ND: Variable ND filter; BS: Beam splitter; OL: Objective lens.

II. Simulating DUV focusing of the AlN metalens

To numerically calculate the reported metalens' focusing capability, a simulation based on the beam propagation method (BPM) was carried out by using a MATLAB module. The metalens was modeled as a field mask, which is a circular aperture with a 250 μm diameter centered on the simulation plane. In the simulation, the metalens plane was divided into Cartesian grids at 0.3 μm resolution. Each grid represents an AlN nanopillar, where the corresponding transmission amplitude and phase were encoded. Within the grid, complex amplitude was assigned, while the region outside the metalens was assigned zero in the amplitude. The incident light was modeled as a plane wave with unit amplitude. The initial field distribution

was obtained by multiplying the incident plane wave with the metalens field mask. The wavefront evolution through the metalens was calculated using the Fast Fourier Transforms (FFT), which solves the scalar wave equation iteratively by applying the Fresnel approximation. The electric field was transformed into the frequency domain via 2D FFT. To model the light propagation along the optical axis (the z-axis in this paper), the longitudinal wavevector k_z was calculated based on the transverse wavevectors k_x and k_y. A phase shift caused by the propagation, described as $e^{ik_z \cdot z}$, was applied to the angular spectrum, and the inverse FFT (IFFT) was used to reconstruct the electric field in the spatial domain for each propagation step. By placing monitors at different positions in the simulation model, the properties of the metalens, such as the focal length, spot size, and the point spread function, can be effectively retrieved.

III. DUV high-NA AlN metalens: simulation demonstration

We simulated the focusing properties of a DUV metalens (NA: 0.8; diameter: 100 μm; focal length: 36 μm) by using commercial software MetaOptic Designer (Synopsys). As the NA increases, the phase gradient ($\frac{\Delta\phi}{\Delta x}$) across the lens surface becomes steeper. The steep phase gradient necessitates smaller unit cell periods (*i.e.*, Δx between the unit cells) to prevent undersampling. **Figure S2a** shows the dependence of the phase and transmittance on the diameter of the pillars (period: 200nm, height: 300nm). The properties of the nanopollars, including the lattice constants, transmittance, and phase, were used in the simulation. In the simulation, the wavelength is 266 nm and polarization is linearly polarized. **Figure S2b** shows the simulation result of DUV focusing by the high-NA metalens. The metalens produced a focal point with a size close to the diffraction limit at z = 62.5 μm. Experimentally implementing the reported high-NA metalenses can be achieved by further optimizing the reported fabrication processes, including improving the exposure and etching parameters in the future.

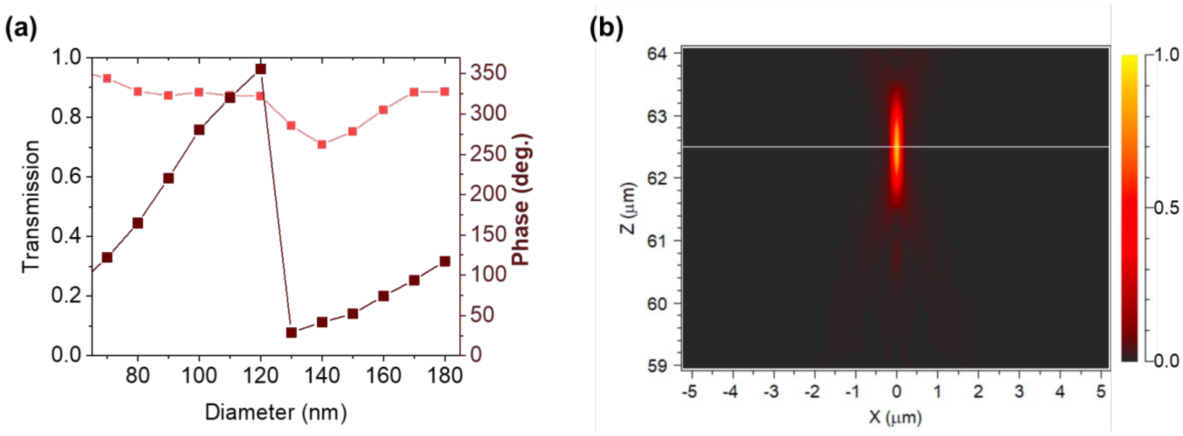

Figure S2. Simulation of high-NA AlN metalens. (a) The parameters of the nanopillars used in the simulation. (b) Focusing profile of the focusing spot generated by the high-NA metalens. The white line shows the position of the focal point.

IV. DUV focusing of the AlN metalens after thermal tretment

Figure S3a shows the focal spot of the AlN metalens after heating at 1000 ˚C for an hour. It shows that metalens maintains proper light-focusing capability after the treatment. The line spread function (**Figure S3b**) and MTF (**Figure S3c**) of the focusing spot slightly change. The FWHM becomes slightly larger than the results in the main text. It could be associated with the change in the morphology of the nanopillars or the contamination from the chemicals in the furnace. Still, the stability of the AlN metalens under high temperatures satisfies general requirements.

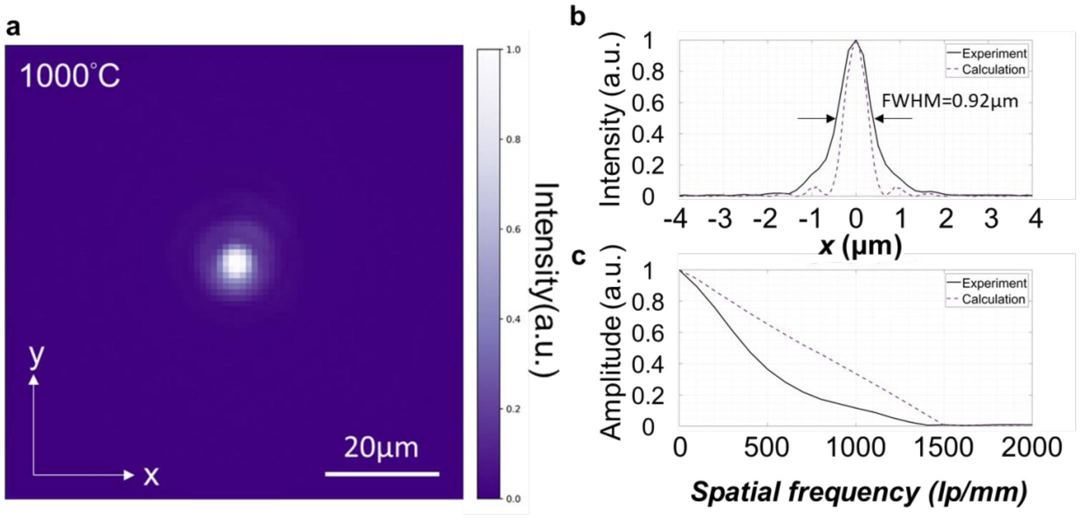

Figure S3. Focusing spot analysis of the metalens after heated at 1000 °C. (a) Image of the focal spot of the heat-treated metalens and the corresponding (b) The line spread function and (c) MTF analysis.

V. SEM image of a laser-produced crater on a photoresist-coated silicon

To clearly observe the structure made by the DUV laser, we adjusted the contrast of the SEM image showed Figure 5 and display it as **Figure S4**. The Si hole diameters made in the Si chip can be identified and indicated by a red arrow (length: 1.5 μm).

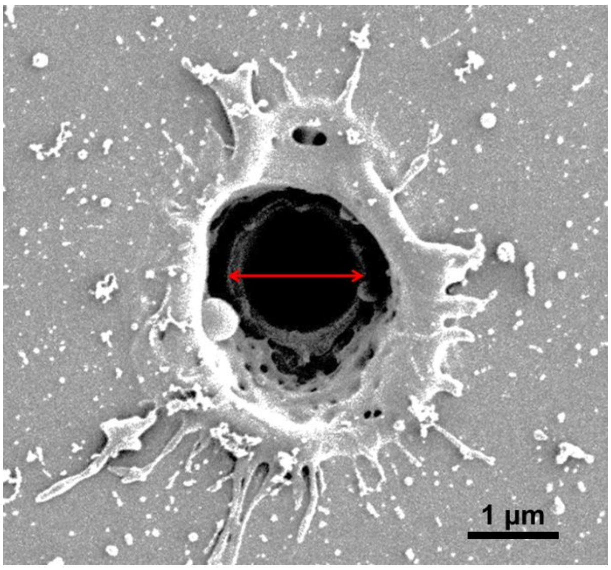

Figure S4. Contrast-adjusted SEM image of a laser-produced crater on a photoresist-coated silicon.

VI. Comparison between the DUV objective and metalens

We compare the performance of an AlN metalens with commercial DUV objectives (LMUL-10X-UVB and LMU-10X-266, both from Thorlabs). To ensure a fair comparison, all components have an NA of 0.25. The metalens has a diameter of 250 μm and a focal length of 484.12 μm. For the objectives, we imported their design files[4, 5] from the Thorlabs website into the OpticStudio (Ansys) simulation software and computed their modulation transfer functions (MTFs). The metalens layout was prepared based on the lens formula detailed in the main text and the nanopillars' simulation data, with its focusing properties simulated using the method described in **Section II**. The results are presented in **Figure S5**.

At $\lambda = 266$ nm, the cutoff spatial frequencies are 1900 lp/mm for the metalens, 1920 lp/mm for the LMUL-10X-UVB objective, and slightly lower at 1840 lp/mm for the LMU-10X-266 objective. Each component has distinct features. For instance, as noted by the supplier, the LMUL-10X-UVB objective provides achromatic focusing across the DUV spectrum. The metalens, on the other hand, is the thinnest device among the three and demonstrates effective light focusing. However, as with similar unit cell designs in our previous work[2, 6], the metalens is expected to exhibit chromatic aberration, leading to a focal shift at different wavelengths.

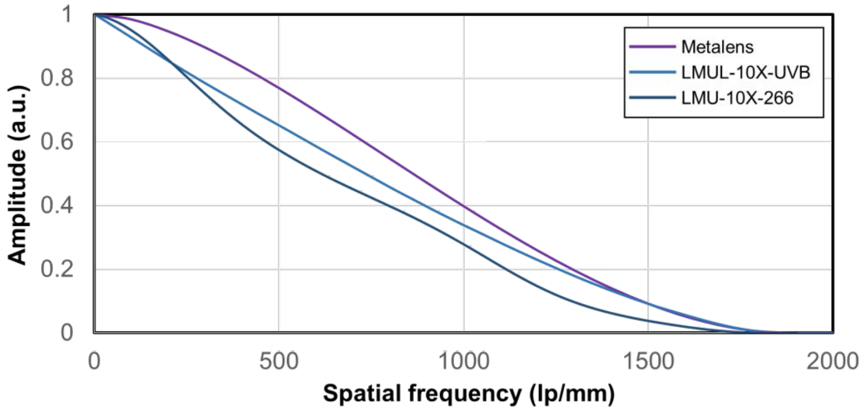

Figure S5. Simulated MTF of the metalens and the objectives. All of the components have an NA of 0.25.

VII. Tolerance of the AlN metalenses for the fabrication imperfections

Fabrication imperfections primarily manifest as nanopillar size deviations, leading to local phase and amplitude errors. To evaluate their impact, we introduced random diameter variations ranging between $\pm d$ to the nanopillars in the metalens layout. This approach simulates a realistic scenario where some nanopillars are larger, and others are smaller than the target size due to fabrication imprecision. Using the data library shown in **Figure 1**, we incorporated these variations into the simulations to analyze the focusing properties of the metalens. The metalens has a diameter of 250 μm and a focal length of 612 μm. **Figures S6a–c**, along with the corresponding zoom-in images, illustrate the distribution of size offsets for ±d = ±5 nm, ±25 nm, and ±50 nm, respectively. The color coding in Figures **S6a-c** represents the local size variations applied to the nanopillars. **Figures S6d-f** show the focal spots generated by metalenses with varying ranges of size offsets, while **Figure S6g** presents the focal spot from a defect-free metalens for comparison. To clearly illustrate the impact of imperfections, all figures are plotted

on a logarithmic scale. The results indicate that although noise is observed, metalenses with imperfections still produce focal spots near the diffraction limit, as shown in the cross-sectional intensity profiles plotted within ±10 μm of the focal spot center (**Figure S6h).** For size offsets of ±5 nm, no significant changes in the focal spot are observed. At ±25 nm, moderate noise becomes noticeable around the focal spot. When the size offset increases to ±50 nm, the noise becomes severe, significantly impacting the focusing quality. The cross-sectional profiles confirm this trend. A comparison of the peak intensity at the focal spot center shows that the metalenses with size offsets of ±5 nm, ±25 nm, and ±50 nm retain 97.7%, 55.6%, and 6.7% of the peak intensity of the defect-free metalens, respectively. According to our SEM analysis, the size offsets in the fabricated nanopillars are less than 15 nm. These simulation results demonstrate that the reported metalens design exhibits a reasonable tolerance to fabrication imperfections.

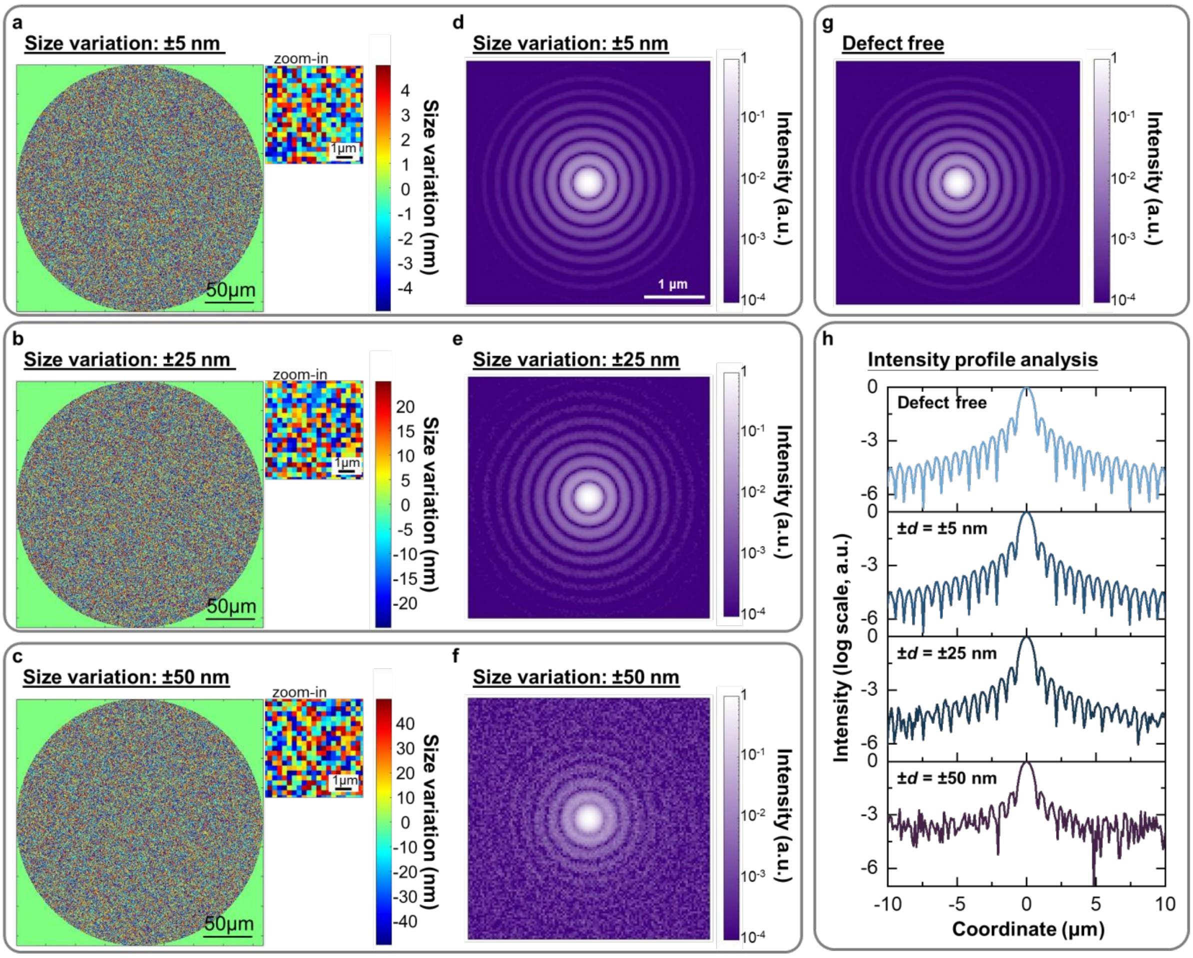

Figure S6. Imperfection tolerance of the AlN metalens. (a)-(c) distribution of size offsets for ±d = ±5 nm, ±25 nm, and ±50 nm, respectively. (d)-(f) The focal spots generated by metalenses with varying

ranges of size offsets. (g) The point spread function generated by the defect-free metalens. (h)The line spread function within ±10 μm of the focal spots' center.

VIII. Fabrication process flow

A 380-nm-thick AlN film was deposited on a c-plane sapphire substrate (**Figure S5**) using plasma-enhanced chemical vapor deposition (PECVD). A silica layer, as the etching mask, was deposited on the AlN surface. We used a standard electron beam lithography process to make the chromium (Cr) etching mask on the top of the sample. Subsequently, we used inductively coupled plasma reactive ion etching (ICP-RIE) to transfer the pattern into the AlN layer and performed wet etching to remove the residual silica layer.

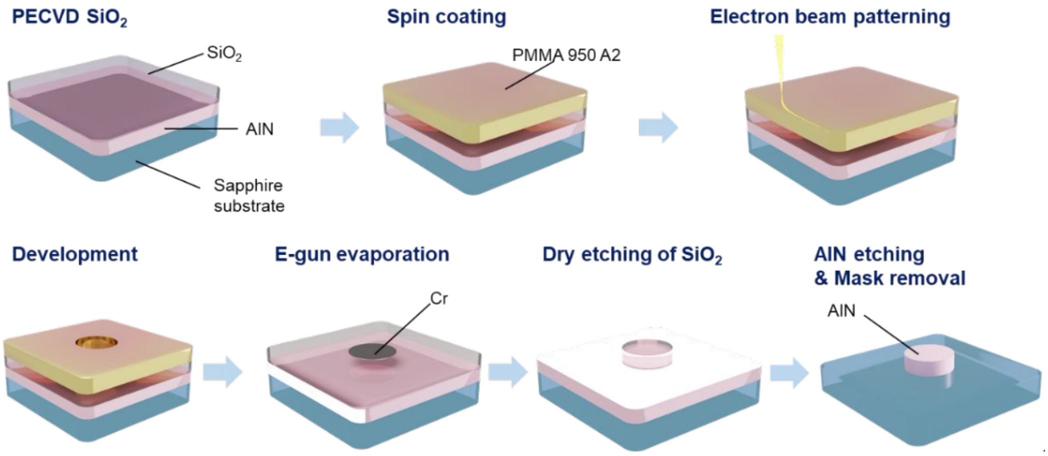

Figure S7. Fabrication process of the AlN metalens.

IX. Surface morphology characterization of the AlN film

Figure S6 presents the AFM image of the AlN film used in this paper. It shows a surface roughness of ~0.6 nm. The flat surface morphology of the film ensures the minimal unwanted surface scattering of DUV light for the fabricated metalenses.

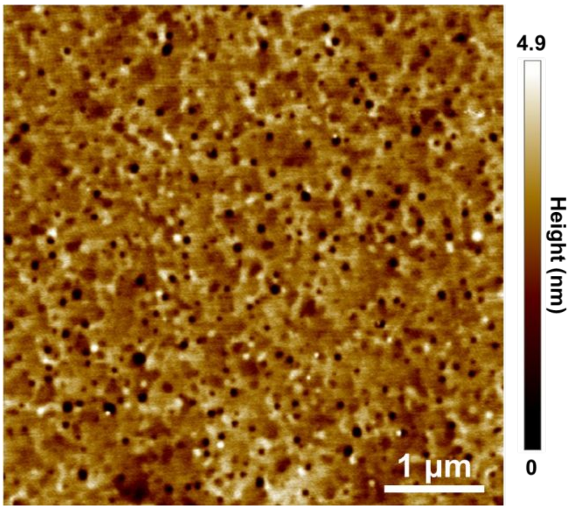

Figure S8. AFM image of the AlN film.

References for Supporting Information:

1. Gu, E.; Choi, H. W.; Liu, C.; Griffin, C.; Girkin, J. M.; Watson, I. M.; Dawson, M. D.; McConnell, G.; Gurney, A. M., Reflection/transmission confocal microscopy characterization of single-crystal diamond microlens arrays. *Appl. Phys. Lett.* **2004,** *84* (15), 2754-2756.
2. Colburn, S.; Zhan, A.; Majumdar, A., Metasurface optics for full-color computational imaging. *Sci Adv* **2018,** *4* (2), eaar2114.
3. Li, Z.; Lin, P.; Huang, Y. W.; Park, J. S.; Chen, W. T.; Shi, Z.; Qiu, C. W.; Cheng, J. X.; Capasso, F., Meta-optics achieves RGB-achromatic focusing for virtual reality. *Sci Adv* **2021,** *7* (5), eabe4458.
4. https://www.thorlabs.com/thorproduct.cfm?partnumber=LMUL-10X-UVB (accessed December 24, 2024).
5. https://www.thorlabs.com/thorproduct.cfm?partnumber=LMU-10X-266 (accessed December 24, 2024).
6. Khorasaninejad, M.; Shi, Z.; Zhu, A. Y.; Chen, W. T.; Sanjeev, V.; Zaidi, A.; Capasso, F., Achromatic Metalens over 60 nm Bandwidth in the Visible and Metalens with Reverse Chromatic Dispersion. *Nano Lett.* **2017,** *17* (3), 1819-1824.
